# Analysis of data of COVID lockdown period: Comorbidity and fatality rates in a few districts of Assam, India

**DOI:** 10.1016/j.dib.2024.110974

**Published:** 2024-09-24

**Authors:** Atlanta Choudhury, Kandarpa Kumar Sarma, Lachit Dutta, Debashis Dev Misra, Aakangkhita Choudhury, Rijusmita Sarma

**Affiliations:** a Department of Electronics and Communication Engineering, Gauhati University, Guwahati, 781014, Assam, India; b Department of Computer Science and Engineering, Faculty of Engineering and Technology, Assam Down Town University, Guwahati 781026, Assam, India; c Department of Microbiology, Jorhat Medical College and Hospital, Jorhat 785001, Assam, India; d Department of Statistics, LCB College, Guwahati 781011, Assam, India

**Keywords:** Comorbidity, COVID-19, Fatality rate, Pandemic, *P*- value

## Abstract

In many regions of the world, significant data collection, analysis, and availability on comorbidity and fatality incidents caused by COVID-19 during the lockdown period (2020–2022) is rare. This is especially true for hospitals and COVID treatment facilities in India. This lack of understanding impedes the development of appropriate treatment options, potentially resulting in inferior planning, patient recovery results, and a load on healthcare resources. This project intends to bridge the gap and enhance patient care in Assam, India, in light of the COVID pandemic. Furthermore, this study aims to determine baseline patient characteristics associated with an elevated risk of death among hospitalized COVID-19 patients in Assam. We employed machine learning (ML) and deep learning (DL) approaches to discover hidden patterns in patient data that could predict which individuals are more sensitive to severe consequences. This knowledge has the potential to transform patient care by allowing doctors to personalize treatment plans and prioritize resources for individuals who are most at risk. A retrospective observational analysis was performed using data from 5329 individuals hospitalized with SARS-CoV-2 illness between April and December 2021. ML and DL algorithms could be used to examine patient characteristics and identify risk factors for death (in this case, 554). We expect this to help us better understand the risk factors for in-hospital death among COVID19 patients in Assam. The findings could be useful in building risk assessment tools to guide patient care.

Specifications Table

**Table:** 

Subject	Health and Medical Sciences
Specific subject area	Data related to death and recovery of patients during COVID lockdown(April 2021 to December,2021)
Type of data	Table, chart, graph
Data collection	The dataset includes 5329 patient data related to COVID-19. COVID-19 patient records with and without comorbidity dataset is a comprehensive collection of patient admission record. Directory includes eight different excel files (with comorbidity, Recovered, Death, without comorbidity, Recovered, Death, mild Covid-19, Severe Covid-19,) The data was sourced from a single source: Gauhati Medical College and Hospital (GMCH), Guwahati and contribute them to an open-source evidence database. It is important to note that the data presented in the manuscript are original and created by the authors themselves. The period of the dataset is April’2021 to Dec’2021.
Data source location	Gauhati Medical College and Hospital (GMCH) in Guwahati, Assam, India Latitude: 26.1549° N Longitude: 91.7643° E
Data accessibility	Repository name: Mendeley Data https://data.mendeley.com/datasets/jck5fv7rdr/1 Data identification number: DOI: 10.17632/9cvpmvkgbb.1 Version 1 Published:16 Sept 2024 Licence: CC BY 4.0 COVID-19 patient records with and without comorbidity
Related research article	To be provided later

## 1 Value of the Data

• Includes data from a wide range of individuals impacted by COVID-19, spanning various age groups, socioeconomic backgrounds, occupations, income levels, education levels, and ethnic identities.
• Can be utilized to examine how these impacts evolved among various identified groups as the pandemic continued and throughout its different phases.
• Performing analysis of death and recovery of patients with co-morbidity and infected with COVID virus: The work discusses the rate of death and recovery of patients suffering from COVID-19 and having certain co-morbidities. The data contains age, gender variations, and pre-recorded medical history. The grouping of data is shown in Fig. 3.
• Diverse scenarios and Demographics: The dataset has varied age groups, and genders and has been collected from different towns in lower Assam, India, making it a comprehensive benchmark for real world scenarios.

## 2 Background

The first COVID-19 case in the Indian state of Assam was reported on March 31, 2020. As of August 19, 2024, the Government of Assam has confirmed a total of 89,468 COVID-19 cases, including 67,641 recoveries, three patient migrations, and 234 deaths in the state. Statistics reveal that 75 % of hospitalized COVID-19 patients have at least one comorbidity. The most prevalent among these are hypertension, diabetes, cancer, neurodegenerative diseases, cardiovascular conditions, obesity, and kidney disorders. Certain comorbidities are known to heighten the risk of severe COVID-19 outcomes [1]. For instance, individuals with particular underlying health conditions are more likely to experience severe effects if they contract the virus.

## 3 Data Description

**Fig. 1:**
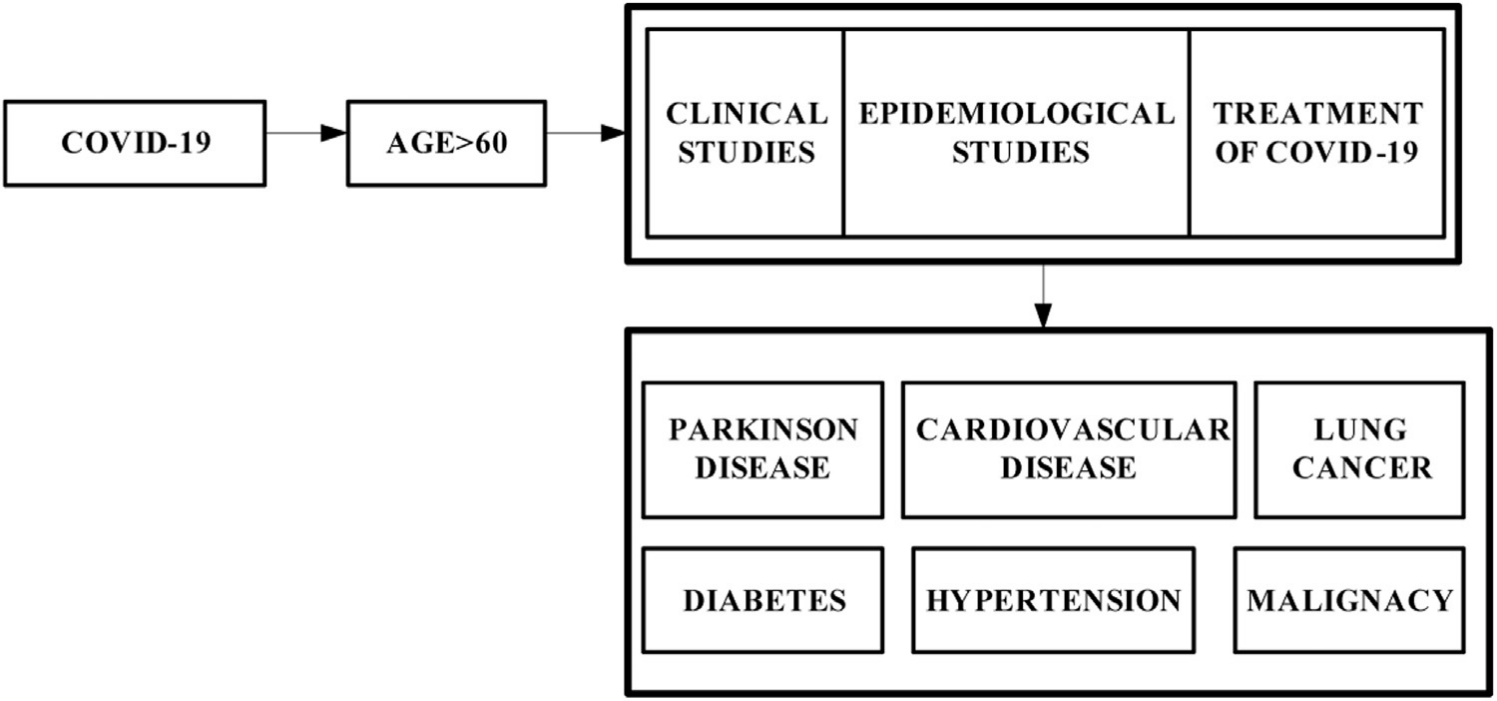
Different classes of comorbidities identified across various studies, encompassing a diverse range of health conditions.

**Fig. 2:**
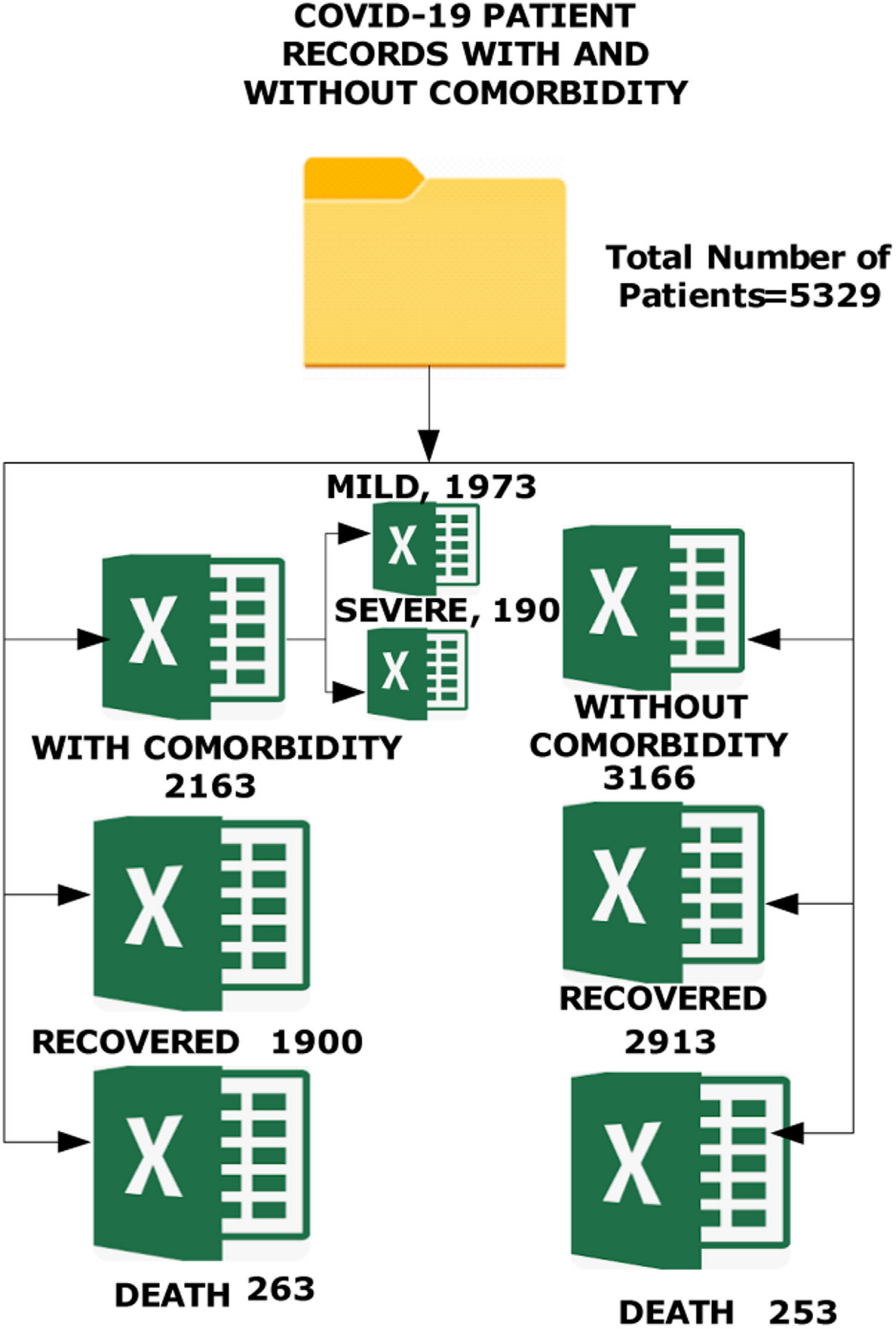
Directory structure of patient records with and without comorbidities.

The objective of the ”Patients with Comorbidity and Fatality Rates” project is to create a high-quality dataset representing various records of patients admitted into Gauhati Medical College and Hospital, Guwahati, Assam, India during the period 1st April 2020 and 31st December 2021 (as shown in Fig. 1). The number of infected individuals with and without comorbidities retrieved and analyzed throughout this period is 5329. Out of these 10.42 % patients have deceased. This dataset is intended to train machine learning (ML) models for accurate prediction of fatality rate during the peak period of COVID-19 [[1], [2], [3]] and [4]. This dataset can be used in current or future pandemic situations along with the trained ML models. Participants’ medical histories and demographic information have been gathered. Symptoms reported by the patients included fever, cough, dyspnea, anosmia, sore throat, diarrhea, headache, myalgia, tiredness, and anorexia [[5], [6], [7]] and [8]. Fasting blood glucose (FBG), liver and renal functions, and COVID-19 biomarkers (total leukocyte count (TLC), neutrophil and lymphocyte counts, and serum levels of C-reactive protein (CRP), D-dimer, ferritin, and lactate dehydrogenase (LDH)) were among the baseline laboratory tests [9,10] and [11] performed. Additionally, arterial blood gases were measured. Chest computed tomography was used to identify COVID-19 pneumonia (CT). Based on the WHO severity categorization, patients were divided into cases classified as severe and non-severe. Fig. 2 presents the hierarchical directory structure of the patient with and without comorbidity dataset, ensuring organized and accessible navigation for researchers and developers. It also displays selected sample data of patient record along with the number of records, offering a clear overview of the dataset's content, quality, and quantity.

## 4 Experimental Design, Materials and Methods

**Fig. 3:**
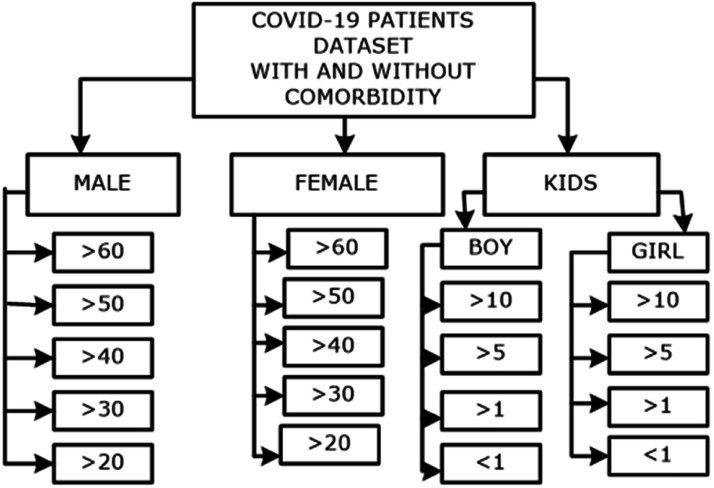
The age distribution and patient count recorded in studies conducted across various districts of Lower Assam.

As was highlighted, this review illustrates the relationship between several comorbidities and COVID-19. A literature search was conducted using several databases and search engines like PubMed to identify such relationships as reported by recent literature. The keywords such as COVID-19, SARS-CoV-2, and comorbidity in COVID-19 were used to get the most relevant articles that support this study. On the other hand, the relationship between Parkinson Disease (PD), cancer, diabetes mellitus, Cardio Vascular Disease (CVD), and hypertension with COVID-19 articles were also used to accomplish this study. Besides, the rest of the published works with mismatched or irrelevant keywords were not considered for this study. All the publications were examined and referenced based on their relevant and compatibility with the current topic of discussion. Although aging is a prominent factor for comorbidities [12], it may not be as labeled as the only confounding factor for the disease severity. Reports have suggested that hospitalized males had the highest mortality rate as compared to females. This association was more prominent with patients with predisposing conditions such as hypertension, diabetes and obesity in an age-dependent manner. One study has shown male patients’ as a predictor of ICU admissions. Contrastingly, in context of the long term COVID-19 manifestations, women were more likely to report uneasiness, breathlessness, and fatigue following recovery. Outcomes in severity also resulted from biochemical differences in males and females; compared to male patients, females had higher lymphocyte counts, higher levels of high-density lipoprotein, as well as lower levels of highly sensitive C-reactive protein. Another hypothesis for gender disparity in protection is that the females have biallelic Toll-like receptor (TLR) 7 expression, thereby leading to a better interferon (IFN)-mediated response after early infection. Interestingly, studies have reported that estrogen confers some protection against the severity of COVID-19. This has been validated in a preclinical setting, where mice infected with the SARS-CoV virus had a higher mortality rate following ovariectomy or estrogen receptor antagonist administration. Fig. 3 presents the age distribution and patient count recorded in studies conducted across various districts of Lower Assam.

Further, pregnant women with mild infection demonstrate same outcome as uninfected pregnant women. However, those with severe infection demonstrate a higher risk of perinatal infection as well as mortality, usually having a tendency to feel unwell and this further exacerbates during COVID-19 infection and may result in worsening of conditions of the patients. Children are disproportionately infected with COVID-19 compared to older population but with low infection severity and could be attributed to lower concentration of ACE2 receptors in children as well as trained/acquired immunity as a result of vaccination, indigenous virus competition, as well as maternal immunity.

Thus, it can be concluded that sex differences play a major role in the outcomes and severity of COVID-19 infection Table 1. Due to the pivotal role of female hormones, they may be less susceptible to long-term manifestations which are prevalent in male patients [6]. The details of patient data recorded during that period is shown in Table 2. The Wilcoxon test is a non-parametric used to verify the mean values of two dependent groups that differ significantly from each other. The p-values for such a series of tests are shown in Table 3, Table 4. The p-values signify the closeness of the outcomes of tests performed using two different datasets [11].

**Table 1:** Patient details (2020–2021).

Sl. no	Month	Confirmed	Recovered	Death	Age	Sex	
						M	F
1	April	576	313	263	43–75	441	135
2	May	1187	976	211	>60	684	503
3	June	596	542	54	43–75	281	314
4	July	790	772	18	60–65	368	422
5	August	645	639	6	80	338	307
6	Sept	583	581	2	65–80	303	297
7	Oct	643	643	–	8–82	403	240
8	Nov	296	296	–		212	84
9	Dec	4	4	–		3	1

**Table 2:** Percentage of patient demise with comorbidity.

Sl.no	Comorbidity	Months									Total Comorbidity	Total Death	Percentage (%)
		April'21	May'21	June'21	July'21	Aug'21	Sept'21	Oct'21	Nov'21	Dec'21			
1	Asthama	1	1	2	8	4	5	7	3	0	31	554	5.60
2	Chronic Kidney Disease										0	554	0.00
3	Chronic Lung Disease (Cough)				1						1	554	0.18
4	High Blood Pressure				1						1	554	0.18
5	Diabetes	1	3	2	1	1	1	0	0	0	9	554	1.62
6	Obesity	0	0	1	0	0	0	0	0	0	1	554	0.18
7	Immunocomprised patient										0	554	0.00
8	Chronic Heart Disease	0	0	1	2	2	0	0	1	0	6	554	1.08
9	chronic neurological Disease										0	554	0.00
10	Chronic Liver Disease										0	554	0.00
11	Cardio Vascular Disease										0	554	0.00
12	Renal Disease Disease										0	554	0.00
13	Malignancy	0	0	1	2	3	0	0	1	0	7	554	1.26
14	anemia	0	0	1	1	1	4	0	0	0	7	554	1.26
15	Parkinson's Disease	0	1	0	0	0	0	0	0	0	1	554	0.18
16	Anxiety Related disorder	0	0	0	0	0	2	0	0	0	2	554	0.36
17	acute Coronary Syndrome	0	0	0	0	0	1	0	0	0	1	554	0.18

**Table 3:** *P*-value calculation derived using Wilcoxon test for Patient data with COVID-19 with and without comorbidities.

Attributes	Patient with COVID-19 with comorbidities		Patient with COVID-19 without comorbidities		*P*-value
Patient Number	*n* = 2163		*n* = 2166		
Age	40–70		45–75		0.012
Sex Male	2909		1590		0.028
Female	746		576		
Temperature	2100		1350		0.081
Heart rate (Beats/min)	1705		1545		0.005
BP (mm Hg)	1450		1300		0.033
**Parameters at the time of admission:**					
1. Fever	1054		1899		0.061
2. Cough	2010		1901		0.044
3. Fatigue	1680		2100		0.002
4. Sore Throat	2150		1705		0.698
5. Diarrhea	1890		1550		0.095
6. SpO_2_	92 %	100	92 %	10	0.046
	93 %	100	93 %	15	0.044
	94 %	200	94 %	20	0.041
	95 %	600	95 %	60	0.038
	96 %	500	96 %	50	0.039
	97 %	1100	97 %	110	0.005
	98 %	1900	98 %	2000	0.061
	99 %	200	99 %	220	0.062
7. Headache	1681		2203		0.423
**Clinical Outcome:**					
1. Discharge	1900		2913		0.011
2. Transfer	652		400		0.131
3. Death	263		253		0.000

**Table 4:** *P*-value calculation derived using Wilcoxon test for Patient data with mild and severe COVID-19.

Comorbidities	Total number of COVID-19 patients with comorbidity (*n* = 2163)	Patient with mild COVID-19 (*n* = 1973)	Patient with severe COVID-19 (*n* = 190)	*P*-value
Asthama	200	100	35	0.000
Chronic Kidney Disease	349	150	70	0.001
Chronic Lung Disease (Cough)	430	175	84	0.005
High Blood Pressure	511	201	95	0.004
Diabetes	520	248	101	0.250
Obesity	315	213	130	0.001
Immune Comprised Patient	120	97	67	0.002
Chronic Heart Disease	420	120	98	0.005
Chronic Neurological Disease	610	155	129	0.168
Chronic Liver Disease	700	220	144	0.265
Cardio Vascular Disease	584	203	123	0.178
Renal Disease	717	133	119	0.156
Malignancy	800	200	35	0.005
Anemia	215	197	59	0.002
Parkinson's Disease	357	219	87	0.198
Anxiety Related Disease	560	80	62	0.011
Acute Coronary Syndrome	445	111	73	0.005
**Comorbidities present**				
One comorbidity	2405	1678	1105	0.000
Two comorbidity	1560	1389	989	0.000
≥ 3 comorbidity	756	545	345	0.000

## Limitations

The retrospective observational analysis of 5329 hospitalized COVID-19 patients in Assam from April to December 2021 has yielded crucial insights into the comorbidities and mortality associated with SARS-CoV-2 during the lockdown period. Utilizing advanced ML and DL techniques, this study has uncovered significant patterns and risk factors that were previously underexplored, revealing those most susceptible to severe outcomes. These findings highlight the urgent need for more extensive data collection and analysis beyond the scope of typical hospital and treatment centre reports. By identifying key baseline traits linked to increased mortality risk, the study provides a foundation for developing more personalized and effective treatment strategies. Such advancements have the potential to enhance patient care, improve recovery rates, and reduce the burden on healthcare resources. Overall, this research makes a significant contribution to the fight against COVID-19, offering valuable insights that can inform better resource allocation and treatment approaches in regions like Assam. It underscores the importance of applying sophisticated analytical methods to deepen our understanding of patient vulnerabilities, ultimately leading to improved outcomes and more efficient management of this global health crisis.

## Ethics Statement

The authors of this dataset, namely Atlanta Choudhury, Kandarpa Kumar Sarma, Lachit Dutta, Debashis Dev Misra, Aakangkhita Choudhury and Rijusmita Sarma, are depicted in the dataset. The authority of GMCH have willingly given consent for their inclusion in the study and have agreed to the public sharing of their data. The authors declare no conflict of interest. This research did not involve animal or human studies and did not inflict harm on any living organism.

## Credit Author Statement

**Atlanta Choudhury:** Conception, Data Collecting, Writing-original-draft, Manuscript Preparation, **Kandarpa Kumar Sarma:** Conception and design, Writing-review-editing, Manuscript correction, Visualization, Supervision, **Lachit Dutta:** Funding acquisition, Manuscript Correction, **Debashis Dev Misra:** Software, Validation, **Aakangkhita Choudhury:** Data Collection, Manuscript Preparation, **Rijusmita Sarma**: Statistical Analysis.

## Data Availability

COVID-19 patient records with and without comorbidity (Original data) (Mendeley Data).

## Appendix Supplementary materials

Image, application 1

## References

[1] Alsaleh M.M., Allery F., Choi J.W., Hama T., McQuillin A., Wu H., &Thygesen J.H (2023). Prediction of disease comorbidity using explainable artificial intelligence and machine learning techniques: a systematic review. Int. J. Med. Inform..

[2] Chatterjee S., Nalla L.V., Sharma M., Sharma N., Singh A.A., Malim F.M., &Khairnar A (2023). Association of COVID-19 with comorbidities: an update. ACS Pharmacol. Transl. Sci..

[3] Patel R., Kooner J.S., Zhang W. (2023). Comorbidities associated with the severity of COVID-19, and differences across ethnic groups: a UK Biobank cohort study. BMC Public Health.

[4] Wei Q., Mease P.J., Chiorean M., Iles-Shih L., Matos W.F., Baumgartner A., &Hadlock J (2023). Risk factors for severe COVID-19 outcomes: a study of immune-mediated inflammatory diseases, immunomodulatory medications, and comorbidities in a large US healthcare system. Medrxiv.

[5] Glancy M., Yeung A., McAuley A., Palmateer N., Bishop J., Taylor B., Hutchinson S. (2024). Factors associated with SARS-CoV-2 testing, diagnosis and COVID-19 disease among individuals prescribed opioid-agonist treatment: a nationwide retrospective cohort study. Clin. Microbiol. Infect..

[6] Khan Z., Mlawa G., Islam S., Elshowaya S., &Saleem M (2024). A retrospective study on the outcome of coronavirus disease 2019 (COVID-19) patients admitted to a district general hospital and predictors of high mortality. Cureus.

[7] Khanna N., Bharti S., Guralarasan G., Kumar T., Sinha R., &Bhadani P.P (2024). Evaluation of comorbidities in the SARS-CoV-2-related mortalities: a retrospective observation from a dedicated COVID-19 care hospital. J. Fam. Med. Prim. Care.

[8] Wright G., Senthil K., Zadeh-Kochek A., Heung-san Au., Zhang J., Huang J., J, Koduri G. (2024). Health-related quality of life after 12 months post discharge in patients hospitalised with COVID-19 related severe acute respiratory infection (SARI): a prospective analysis of SF-36 data and correlation with retrospective admission data on age, disease severity, and frailty. BMJ Open.

[9] Houston K.D., Hartnett J., Rose S.J. (2023). Investigating the association between the COVID-19 vaccination and incident gastrointestinal symptomology: a comprehensive dataset. Data Br..

[10] Jehangir Q., Lee Y., Latack K., Poisson L., Wang D.D., Song S., &Sule A.A (2022). Data of atrial arrhythmias in hospitalized COVID-19 and influenza patients. Data Br..

[11] Guan W.J., Liang W.H., Zhao Y., Liang H.R., Chen Z.S., Li Y.M., He J.X. (2020). Comorbidity and its impact on 1590 patients with COVID-19 in China: a nationwide analysis. Eur. Respir. J..

[12] Djaharuddin I., Munawwarah S., Nurulita A., Ilyas M., Tabri N.A., Lihawa N (2021). Comorbidities and mortality in COVID-19 patients. Gaceta Sanit..

