# Supplementary material for: Analysis of data of COVID lockdown period: Comorbidity and fatality rates in a few districts of Assam, India
Source: Data Brief. 2024 Sep 24;57:110974. doi: 10.1016/j.dib.2024.110974 (PMC11490760; doi:10.1016/j.dib.2024.110974)
Supplement: Supplementary file 1 [file mmc1.pdf]

INSTITUTIONAL ETHICS COMMITTEE
GAUHATI UNIVERSITY

Gauhati University
Assam 781014, India
iec@gauhati.ac.in

31st July, 2023

To,
Professor Kandarpa Kumar Sarma
Department of Electronics and Communication Engineering
Gauhati University

Subject: Decision of GU-IEC on your proposal

Dear Prof. Sarma,

The documentation you submitted, which adhered to the procedures regarding ethical approval for research, has been reviewed. After proper scrutiny of your application, the Committee resolved to approve the proposal to be conducted in its presented form. I'm pleased to inform you that the necessary approval for ethical considerations of the research project has been granted for the duration of the project. If the project is extended, kindly reach out to the Member Secretary of the IEC, GU to request an extension of the ethical approval. You are also asked to report to IEC, GU on the status of the approved study on half yearly basis and to provide a copy of the final report to the IEC, GU at the end of the study.

<u>GU-IEC comments on your proposal</u>

Application No. GUIEC-45/2023, date 31/07/2023

Name of the Applicant: Prof. Kandarpa Kumar Sarma, Department of ECE, GU

Topic: *"Analysis of Data of COVID Lockdown Period: Comorbidity and Fatality Rates in a Few Districts of Assam, India"*

The Committee endorsed the approval subject to submission of the duly signed consent of the participants to be interacted for data collection.

Warm regards,

(Bhaben Tanti)
Member Secretary
